# Mapping data on access to and use of medicines among migrants in Flanders

**DOI:** 10.12688/f1000research.160320.1

**Published:** 2025-02-06

**Authors:** Loes Meukens, Saleh Aljadeeah

**Affiliations:** 1 Department of Public Health, Institute of Tropical Medicine, Antwerp, Flanders, Belgium

**Keywords:** medicine, migrant, Flanders

## Abstract

**Background:**

Migration is a social determinant of health, and migrants often face health inequalities compared to host populations. Migrants are underrepresented in health research in many European countries, including Belgium, which is concerning. The World Health Organization (WHO) developed a comprehensive framework aimed at guiding research on migration and health within the WHO European Region. This initiative supports evidence-based policymaking among European member states by providing a foundational structure for examining various strategies and methodologies. The framework serves as a catalyst for discussion and critical analysis, contributing to the formulation of a global research agenda on migration and health under WHO’s leadership. Additionally, it outlines key research priorities and offers strategic recommendations to enhance the understanding and response to health issues related to migration. One of these recommendations calls on researchers to “maximise the use of existing data from research and routinely collected data in health information systems”.

**Objective:**

The overarching aim of our Datahub initiative is to map available sources of datasets about access to and use of medicines among migrant populations, and test if and under which conditions they can be used in research, by taking the case of Flanders, Belgium.

**Methods:**

This initiative will involve conducting a focused review to map datasets used for reporting access to and use of medicines among migrants, followed by a qualitative study with key informants; a structured analysis of ethical and legal challenges to be addressed when using the datasets we identified for research; and content description and evaluation of the different identified datasets.

**Results:**

We assert that the results of our initiative will help presenting the diverse sources of data about medicines access or use among migrant populations. They will be also used to provide recommendations about enhancing the possibilities of retrieving, and using data, including recommendations for (legal, ethical, methodological) risk mitigation for retrieving and using these data.

**List of abbreviations**
ALLEA: All European Academies
CIOMS: Council for International Organizations of Medical Sciences
EASHW: Ethische Adviescommissie Sociale en Humane Wetenschappen - Ethical Advisory Committee for Social and Human Sciences
GDPR: General Data Protection Regulation
IC(F): Informed Consent (Form)
IRB: Institutional Review Board
ITM: Institute of Tropical Medicine
M&E: Monitoring & Evaluation
NGO: Non-governmental organization
PI: Principal Investigator
WHO: World Health Organization

## 1. Introduction

### 1.1 Background

Migration is a longstanding and growing phenomenon. ^1^ The number of people living outside their countries of origin today is higher than at any time before. ^2^ Migration is a social determinant of health, and migrants often face health inequalities compared to host populations. ^3, 4^ Migrants are underrepresented in health research in many European countries, including Belgium, which is concerning. ^5^ In 2018, The UCL–Lancet Commission on Migration and Health called for more research into migration and health ^6^ as there is an increasing need for data that can reflect the diversity of migrant populations and their diverse health needs. ^5, 7^ Data are collected by different organisations, with different mandates (healthcare facilities, health insurance companies, NGOs, and -perhaps less frequently- research institutes). These data are very scattered.

Globally, the use of medicines stands as the most common used healthcare intervention and is fundamental to preventing, treating, and managing diseases. ^8^ Ensuring fair and consistent access to essential medicines is a critical measure of a nation’s advancement toward achieving Universal Health Coverage. ^9^ However, migrants across various European countries often encounter numerous barriers in their access to medicines. ^10^ Historically, healthcare providers in humanitarian contexts have attempted to overcome these challenges without established systems for managing patient health records. ^11^ This absence of formal medical record systems significantly limits researchers’ ability to systematically assess the quality and access to healthcare services available to migrant populations. Studies exploring the impact of electronic health record (EHR) systems in aiding displaced communities highlight their potential to enhance access to medicines, improve health outcomes, and foster better adherence to medicines. ^11^

### 1.2 Rationale

The WHO presented a framework for migration and health research in the WHO European Region as part of its remit to support evidence-based decision-making in its member states in Europe. ^12^ This framework was designed as a starting point for debating and analysing a broad range of options and approaches to help inform a WHO-led global research agenda on migration and health. The framework offers several recommendations for research, including a call to “maximise the use of existing data from research and routinely collected data in health information systems”. ^12^

Health information systems across Europe often lack comprehensive data on migrant populations, resulting in coverage gap in the data for this populations. ^13^ This issue is not due to limitations in existing knowledge or technological capacity but is primarily shaped by political and ethical considerations. ^13^ A major concern is that categorizing health data by migration status could potentially lead to discrimination against migrant groups. Additionally, collecting and disaggregating data on migrants is viewed as highly sensitive, raising ethical concerns aligned with established guidelines and the European Union’s General Data Protection Regulation (GDPR). ^13, 14^ Nevertheless, the GDPR permits the processing of sensitive data under specific conditions, provided there are legitimate reasons and sufficient protective measures in place. EU data protection regulations do not prohibit the collection and analysis of personal or health data for vulnerable populations, such as migrants, ^13, 15^ Instead, they offer frameworks to mitigate risks related to privacy and confidentiality.

Disaggregating and analyzing health data of migrant populations is essential for gaining a deeper understanding of their distinct healthcare needs and the challenges they face. Without utilizing these data in research, there is a risk of continuing the neglect of migrants’ unique health needs, resulting in inadequate healthcare responses and worsening disparities in access to health services, including essential medicines. ^13, 16– 19^ However, before such data can be effectively used, it is critical to thoroughly consider the ethical implications related to data collection, deidentification, processing, storage, analysis, and dissemination. Implementing strong pseudonymization or anonymization practices, along with secure systems to prevent patient re-identification, is vital to protecting the privacy of migrant populations. Ensuring data protection is particularly important to prevent the misuse of health data for non-health-related purposes, such as immigration enforcement. ^13, 15, 17^

In short, there is a need of a) **mapping** the different datasets that may include data about migrants’ access to and use of medicines; b) understanding how the different datasets can be **deidentified and curated** to ensure data protection, accuracy, completeness and coherence, as well as comparability across datasets; and c) identifying the **methodological and ethical challenges** related to accessing and using these datasets, and addressing these challenges with the ultimate aim of prioritizing the wellbeing and security of migrants. To address these needs, we will use the case of datasets that may contain direct or indirect information about access to medicine for migrants in Flanders, Belgium.

## 2. Study objectives

**The overarching aim** of this proposal is to map the available sources of data about access to and use of medicines among migrant populations in Flanders, Belgium, and assess if and under which conditions they can be used in M&E or research. It is important to recognize that **this project does not aim to actually access data, but get information and guidance on how to access and curate data in a scientifically and ethically sound way**. Any future plan to access and analyse datasets will be the object of a separate protocol.

**The specific objectives are**:

a) To map sources of data that contain information on access to and use of medicines among migrants.
b) To understand how different quantitative and/or qualitative data sources can be de-identified, curated, and assessed for accuracy, completeness and coherence, as well as comparability across datasets, for being used for M&E or research.
c) To identify and describe the challenges related to accessing and using these data, including ethical and legal challenges, and to develop and describe viable strategies and recommendations for deidentification, data curation and risk mitigation.
d) To assess the reliability and feasibility of applying the sources of data identified by this study in future research studies that aim at evaluating the access of/use of medicines among the diverse migrants population.

## 3. Study design

**Figure 1.**
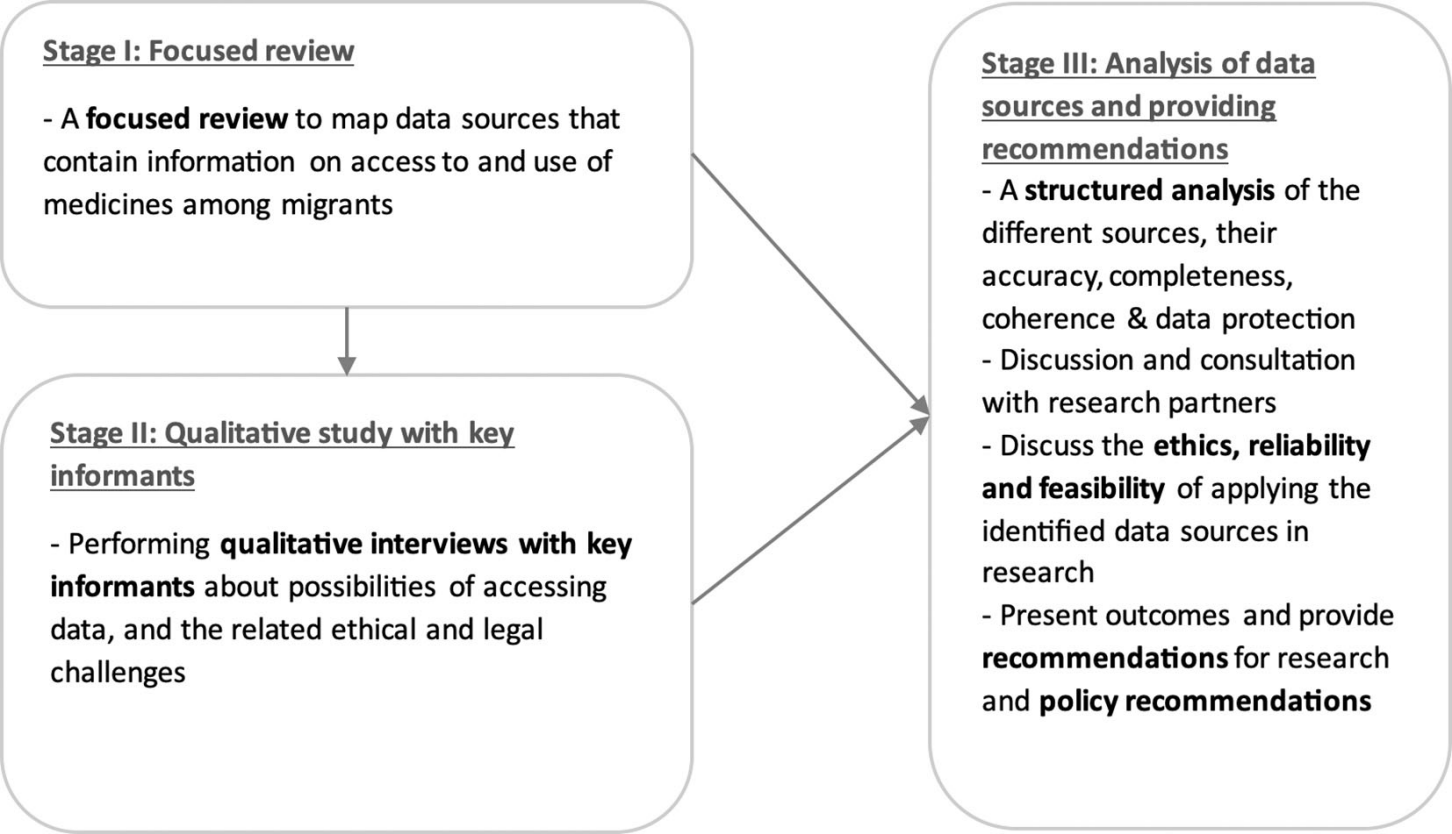
The overall sequential design of the research study, fractionated in three stages.

This project includes three separate stages ( Figure 1). During **stage I**, a focused review will be performed to collect information on different data sources that were used in published literature and reports in the relevant field. The outcome of a recently completed scoping review “Access to medicines among asylum seekers, refugees, and undocumented migrants across the migratory cycle in the European Union, European Economic Area, Switzerland and the UK: a scoping review” ^20^ led by the PI, which focused on access to medicines among migrants in Europe, will inform the focused review. **Stage II** will consist of qualitative interviews of key-informants in Flanders to discuss the possibilities of accessing, deidentifying and curating data, and of the ethical and legal challenges related to accessing and using these data. Key informants are a) those who are working in the collection, management and processing of datasets related access to or use of medicine (we will refer to them as data gatekeepers), and b) members of migrant communities. With **stage III**, we will discuss the reliability and feasibility of applying the identified data sources in scientifically and ethically sound research. This stage will imply triangulating the outcomes of Stage I and II, and formulating policy recommendations as well as recommendations for further research.

## 4. Methods

### 4.1 Procedures

**4.1.1 Focused review**:

We will perform a focused review to identify data sources that have been used in research and grey literature to report on access to medicine among migrants in Belgium. For this focused review we will follow the following steps:

a. Defining the scope and objectives: We will search for studies and grey literature sources that reported on access to medicine among migrants in two regions of Belgium: Flanders and Brussels.
b. Defining eligibility criteria: We will include studies and grey literature sources that have reported on access to and use of medicines among migrant populations in English and Dutch and was published after 2010.
c. Defining data Sources: We will also conduct our search on PubMed, Google Search, and websites of relevant governmental and non-governmental organisations, e.g. WHO, Médecins Sans Frontières, Médecins du Monde, Sciensano, FOD Volksgezondheid, Vlaams Agentschap Zorg en Gezondheid.
d. Screening and selecting materials: We will screen titles and abstracts against our inclusion criteria to identify potentially relevant documents. This will be followed by full text screening.
e. Data Extraction and Synthesis: We will extract data on the sources of data used in the studies and reports. In addition, we will extract the information about the challenges related to accessing, collecting and using these data for research. The identified common sources of data will be used for shaping the qualitative interviews in stage II.

**4.1.2 Qualitative study**:

Qualitative interviews will be conducted with key informants, i.e. data gatekeepers and migrant community members. Details are separately explained in chapters 4.2-4.4.

**4.1.3 Triangulation of the outcomes of the first two steps**:

We will compare the outcomes of the focused review (stage I) with the results of the qualitative interviews (stage II) highlighting areas of convergence, divergence, or expansion. The compared and contrasted outcomes of both studies will be presented and discussed with a research advisory group, comprising a group of researchers from the ITM (Karina Kielmann, Joris Michielsen, Houssynatou Sy, Mira Schneider, and Marie Meudec) and from Boston University (Veronika Wirtz), to broaden the understanding of the outcomes and formulate recommendations for further research. Table 1 lists a summary of the different stages that will take place during the study and the respective methods for data collection. More details are explained in chapter 4.

**Table 1.** Summary of project research objectives, questions, methods and logical sequence across the project stages.

Stages	Research objectives	Research questions	Methods and data sources
**I**	a- To map secondary data sources that contain information on access to and use of medicines among migrants.	Which sources/organizations in Flanders gather data on access to and use of medicines among migrants in Flanders?	A focused review to map datasets used for reporting access to and use of medicines among migrants
**II**	b- To understand how these different data sources can be accessed, deidentified and curated, and identify the methodological and ethical challenges related to accessing and using these data.	What are the challenges and facilitators of ethically accessing data on medicines access/use among migrants in Flanders?	Qualitative interviews with key informants Thematic analyses
**III**	c- To discuss the reliability and feasibility of applying the identified data sources in research studies that aim of evaluating the access to/use of medicines among migrants population in Flanders.	How can these data be used in future research studies that aim of evaluating the access to/use of medicines among the migrants population in Flanders?	Content description and evaluation of the different identified data sources, including discussion and consultation with research partners

### 4.2 Study population

During stage II, qualitative interviews will be performed in order to obtain insights from community members and data gatekeepers in the use of data sources regarding access to and use of medicines among migrants in Flanders.

**4.2.1 Inclusion criteria**:

• Adult (≥ 18 years).
• **Community members:** migrants who have been living in Belgium for over 5 years and are active members of the community. These include individuals with migration background who are in leadership roles within community organizations and social clubs.
• **Data gatekeepers**: representatives of NGOs that provide care for migrants, staff of insurance companies, administrative and health staff in healthcare facilities (hospitals, outpatient clinics, pharmacies) in charge of collecting or processing data regarding access to or use of medicines, and other categories potentially identified through snowballing.
• Willing and able to provide written informed consent.

**4.2.2 Exclusion criteria**:

• Eligible persons who decline to consent to participation.
• Minors.

**4.2.3 Recruitment and sampling**:

Contacts from the social and professional network of the principal investigator (PI), co-investigators and scientific advisors will be purposively sampled, aiming at enrolling a diverse group with a broad range of experiences and perspectives. Snowball sampling will be used to identify other potential participants, for reaching out to more isolated groups. Given the diversity of the data sources, we will likely interview a higher number of the data gatekeepers in order to improve balance in including different groups of participants.

### 4.3 Data collection

Data will be collected by the Principal Investigator (SA) or co-investigator (LM) remotely via GDPR-compliant online platforms, or -preferably- by face-to-face interviews. Data will be collected through semi-structured qualitative interviews, conducted in English or Dutch, based on the preference of the participant.

It is estimated that approx. 10-20 participants will be needed. However, the exact sample size will depend on the quality and diversity of the data; recruitment will continue and data will be collected until saturation. Topic guides by categories are being developed. A draft version is submitted, which will be finalized in line with the findings of the mapping in Stage I. ^22^

Remote interviews will be conducted via European General Data Protection Regulation (GDPR)-compliant platforms (e.g. Teams), and according to participant’s preference. Face-to-face interviews will be conducted in a secure setting proposed by/agreed with the interviewee. The interview sessions (remote and in-person) will be audio-recorded, if the participant explicitly agrees to be recorded; otherwise, hand notes will be taken by the Principal Investigator or co-investigator.

### 4.4 Qualitative data analysis

The recordings will be transcribed, and translated into English when needed. Recordings will be deleted once transcription and cleaning are completed. No direct personal identifiers will be transcribed, including the name of the employer or names of third parties who could be mentioned during the interview. Word will be used for data transcription. Following the thematic content analysis approach suggested by Braun and Clarke, ^21^ data will be coded and categorized and themes will be identified. This will involve collating codes into potential themes and gathering all data relevant to each potential theme.

## 5. Results

A study report will be shared with study participants through the same channels that were used to conduct the interviews with them, if they agreed to be further contacted by email for this scope. The study findings will form the basis for one or more open-access scientific publication(s) in a peer-reviewed journal. The outcomes will be also disseminated by means of a policy brief, conference presentations and meetings of NGOs active in assistance to migrants in Flanders or other relevant stakeholders who are involved in migrant assistance, healthcare or administrative affairs in Flanders. The policy briefs and dissemination to stakeholders are key for maximizing the social value of the study, and the likelihood that findings may provide useful guidance to improve the collection and use in research of data on migrants health.

## Authors’ contributions

SA contributed to conceptualisation of the study, article selection, data collection through semi-structured qualitative interviews, data analysis, protocol drafting and critical revisions. LM contributed to article selection, data collection through semi-structured qualitative interviews, data analysis, protocol drafting and critical revisions.

## Ethics review

The protocol was submitted for formal review and approval to the Institutional Review Board (IRB) of the Institute of Tropical Medicine [Approval number 1767/24; Date of approval 02/05/2024].

The study will be carried out according to the principles stated in the Declaration of Helsinki (2013 and any further revisions).

Informed Consent (IC) was obtained prior to interviews. Study participants (adults only) were informed that participation in the study is completely voluntary and that the participant can withdraw from the study at any time without any negative consequences.

The interviewer provided all the information about the study, and went through the Participant Information Sheet together with the participant in advance. The Data Protection Officer contact details were included in the ICF for questions about personal data processing.

## Data Availability

No data are associated with this article. Figshare: Interviews topic guides_ENG (version 2).pdf, https://doi.org/10.6084/m9.figshare.28219859.v1. ^22^ This project contains the following underlying data:
• Interviews topic guides_ENG (version 2).pdf Data are available under the terms of the Creative Commons Attribution 4.0 International license (CC-BY 4.0).
