# Mapping data on access to and use of medicines among migrants in Flanders

**DOI:** 10.12688/f1000research.160320.2

**Published:** 2025-07-08

**Authors:** Loes Meukens, Saleh Aljadeeah

**Affiliations:** 1 Department of Public Health, Institute of Tropical Medicine, Antwerp, Flanders, Belgium

**Keywords:** medicine, migrant, Flanders

## Abstract

**Background:**

Migration is a social determinant of health, and migrants often face health inequalities compared to host populations. Migrants are underrepresented in health research in many European countries, including Belgium, which is concerning. The World Health Organization (WHO) developed a comprehensive framework aimed at guiding research on migration and health within the WHO European Region. This study supports evidence-based policymaking among European member states by providing a foundational structure for examining various strategies and methodologies. The framework serves as a catalyst for discussion and critical analysis, contributing to the formulation of a global research agenda on migration and health under WHO’s leadership. Additionally, it outlines key research priorities and offers strategic recommendations to enhance the understanding and response to health issues related to migration. One of these recommendations calls on researchers to “maximise the use of existing data from research and routinely collected data in health information systems”.

**Objective:**

The overarching aim of our study is to map available sources of datasets about access to and use of medicines among migrant populations, and test if and under which conditions they can be used in research, by taking the case of Flanders, Belgium.

**Methods:**

This study will involve conducting a focused review to map datasets used for reporting access to and use of medicines among migrants, followed by a qualitative study with key informants; a structured analysis of ethical and legal challenges to be addressed when using the datasets we identified for research; and content description and evaluation of the different identified datasets.

**Results:**

We assert that the results of our study will help presenting the diverse sources of data about medicines access or use among migrant populations. They will be also used to provide recommendations about enhancing the possibilities of retrieving, and using data, including recommendations for (legal, ethical, methodological) risk mitigation for retrieving and using these data.

**List of abbreviations**
ALLEA: All European Academies
CIOMS: Council for International Organizations of Medical Sciences
EASHW: Ethische Adviescommissie Sociale en Humane Wetenschappen - Ethical Advisory Committee for Social and Human Sciences
GDPR: General Data Protection Regulation
IC(F): Informed Consent (Form)
IRB: Institutional Review Board
ITM: Institute of Tropical Medicine
M&E: Monitoring & Evaluation
NGO: Non-governmental organization
PI: Principal Investigator
WHO: World Health Organization

## 1. Introduction

### 1.1 Background

Migration is a longstanding and growing phenomenon. ^1^ The number of people living outside their countries of origin today is higher than at any time before. ^2^ Migration is a social determinant of health, and migrants often face health inequalities compared to host populations. ^3, 4^ Migrants are underrepresented in health research in many European countries, including Belgium, which is concerning. ^5^ In 2018, The UCL–Lancet Commission on Migration and Health called for more research into migration and health ^6^ as there is an increasing need for data that can reflect the diversity of migrant populations and their diverse health needs. ^5, 7^ Data are collected by different organisations, with different mandates (healthcare facilities, health insurance companies, NGOs, and -perhaps less frequently- research institutes). However, the collection of such data remains fragmented across settings and time periods, often lacking standardised and shared procedures. This impedes efforts to monitor and improve migrant health in Europe. ^8^ As Lebano et al. ^13^ concluded in their scoping review, the lack of reliable and routine data collection, inconsistent definitions of migrant categories, and variations in national health systems all present major barriers to assessing and comparing health outcomes. ^8^ While interest in migrants’ health is growing, these limitations create serious challenges in developing evidence-based policies and tailored healthcare interventions. ^8^

Globally, the use of medicines stands as the most common used healthcare intervention and is fundamental to preventing, treating, and managing diseases. ^8^ Ensuring fair and consistent access to essential medicines is a critical measure of a nation’s advancement toward achieving Universal Health Coverage. ^9^ However, migrants across various European countries often encounter numerous barriers in their access to medicines. ^10^ Historically, healthcare providers in humanitarian contexts have attempted to overcome these challenges without established systems for managing patient health records. ^11^ This absence of formal medical record systems significantly limits researchers’ ability to systematically assess the quality and access to healthcare services available to migrant populations. Studies exploring the impact of electronic health record (EHR) systems in aiding displaced communities highlight their potential to enhance access to medicines, improve health outcomes, and foster better adherence to medicines. ^11^

Access to essential medicines for migrants in Europe varies significantly across countries due to differences in healthcare systems, legal frameworks, and the socio-political status of migrants. Migrants, particularly asylum seekers, refugees, and undocumented migrants, often face substantial barriers to accessing medicines. These barriers include limited legal entitlements, financial constraints, language issues, and lack of familiarity with healthcare systems. ^12^ In many EU countries, asylum seekers and undocumented may be excluded from national health insurance systems or face long waiting periods for coverage, which can delay or restrict access to essential medications. ^13^ In Belgium, asylum seekers are entitled to medical care under the “Aide Médicale Urgente” (AMU) system, which covers urgent and necessary healthcare, including some essential medicines. However, this provision often does not cover the full range of chronic disease treatments. ^14^ While some NGOs and local health actors provide support to fill these gaps, persistent challenges remain, underscoring the need for more inclusive and coordinated policies and better data to ensure equitable access to medicines for all migrant populations. ^15^

### 1.2 Rationale

The WHO developed a framework for migration and health research in the WHO European Region to support evidence-based decision-making in its member states. ^16^ This framework emphasizes the importance of maximizing the use of existing data from research and routinely collected data in health information systems”. ^16^

Health information systems across Europe often lack comprehensive data on migrant populations, resulting in gaps in the data for these populations. ^17^ A concern is that categorizing health data by migration status could potentially lead to discrimination against migrant groups. Additionally, collecting and disaggregating data on migrants is viewed as highly sensitive, raising ethical concerns aligned with established guidelines and the European Union’s General Data Protection Regulation (GDPR). ^17, 18^ However, the GDPR permits the processing of sensitive data under specific conditions, provided there are legitimate reasons and sufficient protective measures in place. EU data protection regulations do not prohibit the collection and analysis of personal or health data for vulnerable populations, such as migrants. ^17, 19^ These regulations offer frameworks to mitigate risks related to privacy and confidentiality.

Disaggregating and analyzing health data of migrant populations is essential to understanding of their distinct healthcare needs and the challenges they face. Without utilizing these data, there is a risk of neglecting migrants’ unique health needs, resulting in inadequate healthcare responses and worsening disparities in access to health services, including essential medicines. ^17, 20– 23^ Before such data can be effectively used, ethical implications regarding data collection, deidentification, processing, storage, analysis, and dissemination. Implementing strong pseudonymization or anonymization practices, along with secure systems to prevent patient re-identification, must be considered. Protecting privacy is particularly important to prevent the misuse of health data for non-health-related purposes, such as immigration enforcement. ^17, 19, 21^

In short, this study aims to a) **mapping** the different datasets that may include data about migrants’ access to and use of medicines; b) understanding how the different datasets can be **deidentified and curated** to ensure data protection, accuracy, completeness and coherence, as well as comparability across datasets; and c) identifying the **methodological and ethical challenges** related to accessing and using these datasets, and addressing these challenges with the ultimate aim of prioritizing the wellbeing and security of migrants. The WHO framework calls for maximizing the use of existing data, which directly aligns with this study’s goal of mapping datasets that may contain information on migrants’ access to and use of medicines in Flanders, Belgium. This approach will inform data-driven policies, enabling more effective healthcare responses for migrant populations in Belgium. To address these needs, we will use the case of datasets that may contain direct or indirect information about access to medicine for migrants in Flanders, Belgium. We focused on Flanders due to pragmatic reasons related to the language capacity of our research team, and our established network in the region. Expanding to other regions of Belgium would have introduced logistical challenges, including language barriers, and increased costs and time constraints. We acknowledge that this regional focus limits the generalizability of our findings.

## 2. Study objectives

**The overarching aim** of this proposal is to map the available sources of data about access to and use of medicines among migrant populations in Flanders, Belgium, and assess if and under which conditions they can be used in M&E or research. It is important to recognize that **this project does not aim to actually access data, but get information and guidance on how to access and curate data in a scientifically and ethically sound way**. Any future plan to access and analyse datasets will be the object of a separate protocol.

**The specific objectives are**:

A. Data mapping: This objective focuses on identifying existing datasets related to migrant medicine use in Flanders. We will assess the availability and coverage of these sources, considering whether they can be used for research and M&E purposes.
B. Feasibility assessment: Our study will evaluate feasibility of applying the sources of data identified by this study in future research studies that aim at evaluating the access of/use of medicines among the diverse migrant population.
C. Ethical considerations: We will describe the challenges related to accessing and using these data, including ethical and legal challenges, and to develop and describe viable strategies and recommendations for deidentification, data curation and risk mitigation.

## 3. Study design

**Figure 1.**
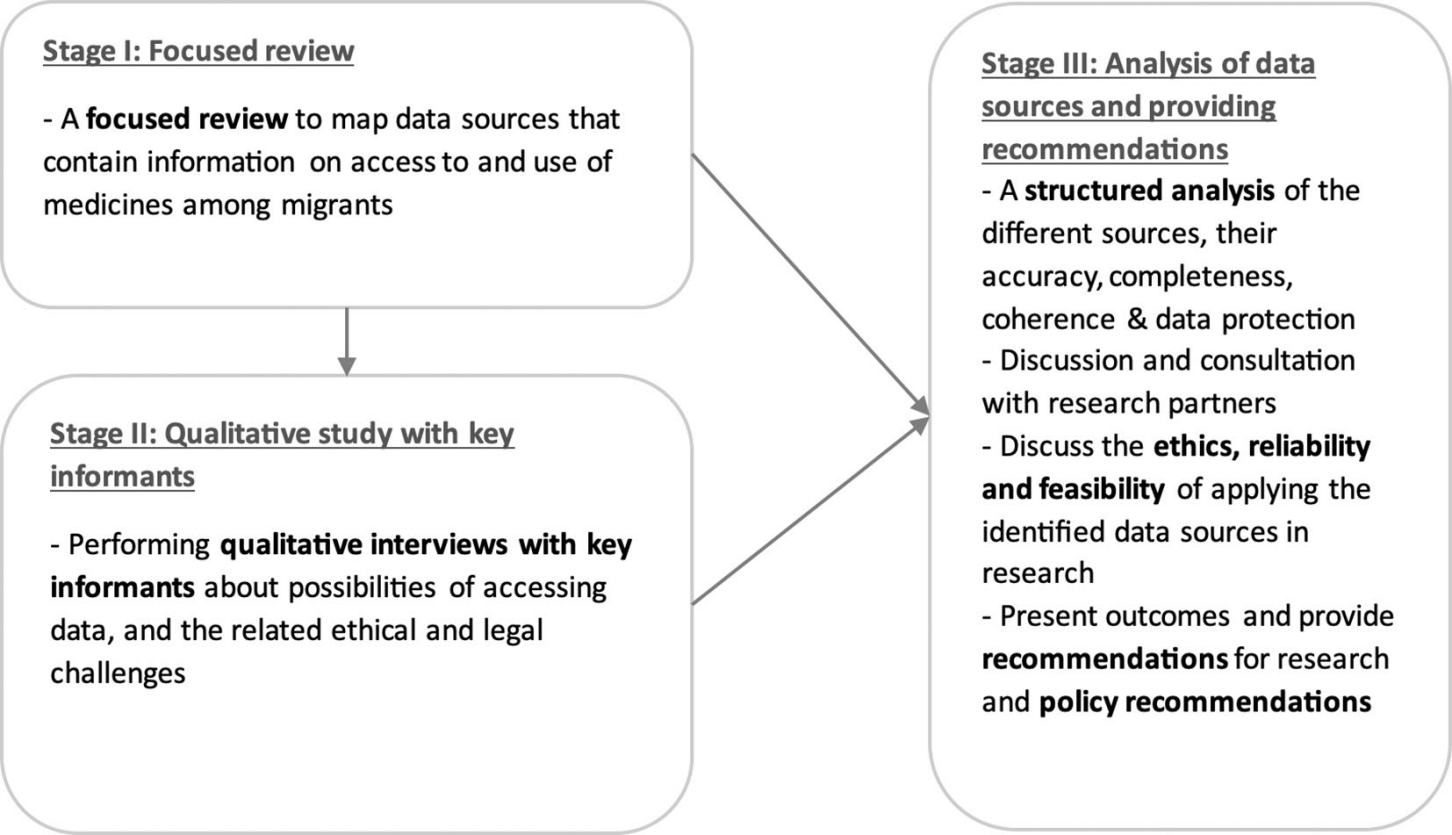
The overall sequential design of the research study, fractionated in three stages.

This project includes three separate stages ( Figure 1). During **stage I**, a focused review will be performed to collect information on different data sources that were used in published literature and reports in the relevant field. The outcome of a recently completed scoping review “Access to medicines among asylum seekers, refugees, and undocumented migrants across the migratory cycle in the European Union, European Economic Area, Switzerland and the UK: a scoping review” ^24^ led by the PI, which focused on access to medicines among migrants in Europe, will inform the focused review. **Stage II** will consist of qualitative interviews of key-informants in Flanders to discuss the possibilities of accessing, deidentifying and curating data, and of the ethical and legal challenges related to accessing and using these data. Key informants are a) those who are working in the collection, management and processing of datasets related access to or use of medicine (we will refer to them as data gatekeepers), and b) members of migrant communities. With **stage III**, we will discuss the reliability and feasibility of applying the identified data sources in scientifically and ethically sound research. This stage will imply triangulating the outcomes of Stage I and II, and formulating policy recommendations as well as recommendations for further research.

## 4. Methods

### 4.1 Procedures

**4.1.1 Focused review**:

We will perform a focused review to identify data sources that have been used in research and grey literature to report on access to medicine among migrants in Belgium. For this focused review we will follow the following steps:

a. *Defining the scope and objectives:* We will search for studies and grey literature sources that reported on access to medicine among migrants in two regions of Belgium: Flanders and Brussels.
b. *Defining eligibility criteria:* We will include studies and grey literature sources that have reported on access to and use of medicines among migrant populations in English and Dutch and was published after 2010.
c. *Defining data Sources:* We will also conduct our search on PubMed, Google Search, and websites of relevant governmental and non-governmental organisations, e.g. WHO, Médecins Sans Frontières, Médecins du Monde, Sciensano, FOD Volksgezondheid, Vlaams Agentschap Zorg en Gezondheid.
d. *Screening and selecting materials:* We will screen titles and abstracts against our inclusion criteria to identify potentially relevant documents. This will be followed by full text screening.
e. *Data Extraction and Synthesis:* We will extract data on the sources of data used in the studies and reports. In addition, we will extract the information about the challenges related to accessing, collecting and using these data for research. The data extracted of this focused review will be collated, summarized and reported in a manner that aligns with the aim of the focused review. We will conduct hematic analysis and present its outcomes in a narrative format. The identified common sources of data will be used for shaping the qualitative interviews in stage II.

**4.1.2 Qualitative study**:

Qualitative interviews will be conducted with key informants, i.e. data gatekeepers and migrant community members. Details are separately explained in chapters 4.2-4.4.

**4.1.3 Triangulation of the outcomes of the first two steps**:

We will compare the outcomes of the focused review (stage I) with the results of the qualitative interviews (stage II), highlighting areas of convergence, divergence, or expansion. To enable meaningful triangulation, we will compare the types and characteristics of data sources identified in Stage I (e.g., scope, accessibility, population coverage, variables collected) with insights gathered in Stage II about their practical use, availability, and limitations as described by the participants. In particular, we will assess whether data sources identified in literature are also recognised or used by key stakeholders in Flanders, and whether additional data sources are known to local actors but were reported in the literature. We will also compare reported legal and ethical considerations across both stages. This comparison will help to validate, expand, or challenge the findings from the literature and identify gaps between formal reporting and actual data access and usage practices in the field. The compared and contrasted outcomes of both studies will be presented and discussed with a research advisory group, to broaden the understanding of the outcomes and formulate recommendations for further research. Table 1 lists a summary of the different stages that will take place during the study and the respective methods for data collection.

**Table 1.** Summary of project research objectives, questions, methods and logical sequence across the project stages.

Stages	Research objectives	Research questions	Methods and data sources
**I**	a- To map secondary data sources that contain information on access to and use of medicines among migrants.	Which sources/organizations in Flanders gather data on access to and use of medicines among migrants in Flanders?	A focused review to map datasets used for reporting access to and use of medicines among migrants
**II**	b- To understand how these different data sources can be accessed, deidentified and curated, and identify the methodological and ethical challenges related to accessing and using these data.	What are the challenges and facilitators of ethically accessing data on medicines access/use among migrants in Flanders?	Qualitative interviews with key informants Thematic analyses
**III**	c- To discuss the reliability and feasibility of applying the identified data sources in research studies that aim of evaluating the access to/use of medicines among migrants population in Flanders.	How can these data be used in future research studies that aim of evaluating the access to/use of medicines among the migrants population in Flanders?	Content description and evaluation of the different identified data sources, including discussion and consultation with research partners

### 4.2 Study population

During stage II, qualitative interviews will be performed in order to obtain insights from community members and data gatekeepers in the use of data sources regarding access to and use of medicines among migrants in Flanders.

**4.2.1 Inclusion criteria**:

• Adult (≥18 years).
• **Community members:** Migrants who have been living in Belgium for over 5 years and are active members of the community. These include individuals with migration background who are in leadership roles within community organizations and social clubs.
• **Data gatekeepers**: Representatives of NGOs that provide care for migrants, staff of insurance companies, administrative staff and healthcare practitioners in healthcare facilities (hospitals, outpatient clinics, pharmacies) in charge of collecting or processing data regarding access to or use of medicines, representatives of public health offices or institutions, and other categories potentially identified through snowballing.
• Willing and able to provide written informed consent.

**4.2.2 Exclusion criteria**:

• Eligible persons who decline to consent to participation.
• Minors.

**4.2.3 Recruitment and sampling**:

Contacts from the social and professional network of the principal investigator (PI), co-investigators and scientific advisors will be purposively sampled, aiming at enrolling a diverse group with a broad range of experiences and perspectives, for both key informants and community members. Data gatekeepers will primarily be approached through institutional networks, professional contacts, and health sector organizations. In contrast, community members will be approached via civil society organizations, community-based networks, and local NGOs working with migrant populations. Snowball sampling will be used to identify other potential participants, for reaching out to more isolated groups. Given the diversity of the data sources, we will likely interview a higher number of the key informants in order to improve balance in including different groups of participants.

### 4.3 Data collection

The interviews will collect two types of data. From data gatekeepers, we will collect information on the availability, accessibility, structure, content, and quality of existing data sources relevant to access to and use of medicines among migrants in Flanders, as well as legal, technical, and ethical considerations in using these data. From members of migrant communities, we will collect insights on whether and how their access to medicines may be represented (or overlooked) in existing data sources, including perceptions of data use, trust in institutions, and barriers to inclusion.

Data will be collected by the Principal Investigator (SA) or co-investigator (LM) remotely via GDPR-compliant online platforms, or -preferably- by face-to-face interviews. Data will be collected through semi-structured qualitative interviews, conducted in English or Dutch, based on the preference of the participant. Face-to-face interviews will be conducted in a secure setting proposed by/agreed with the interviewee. The interview sessions (remote and in-person) will be audio-recorded, if the participant explicitly agrees to be recorded; otherwise, hand notes will be taken by the Principal Investigator or co-investigator. In the Stage I of the study, when performing the focused review, no personal data will be processed. For the Stage II, when conducting the interviews, the lawful ground for the processing of the participant personal data will be the written consent of the participant. The study will also be entered in the Institute of Tropical Medicine GDPR registry of personal data processing activities and the requirements are reviewed by the Data Protection Officer. All study data will be pseudonymized at the earliest convenience. Identifiers are retained at a separate and secure location, and deleted (full anonymization) after the study is published. Identifiable information in any audio or videocall recording will also be minimized to the extent possible.

It is estimated that approx. 10-20 participants will be needed. However, the exact sample size will depend on the quality and diversity of the data; recruitment will continue and data will be collected until saturation. Topic guides by categories are being developed. A draft version is submitted, which will be finalized in line with the findings of the mapping in Stage I. ^26^

### 4.4 Qualitative data analysis

The recordings will be transcribed, and translated into English when needed. Recordings will be deleted once transcription and cleaning are completed. No direct personal identifiers will be transcribed, including the name of the employer or names of third parties who could be mentioned during the interview. Word will be used for data transcription. Following the thematic content analysis approach suggested by Braun and Clarke, ^25^ data will be coded and categorized and themes will be identified. This will involve collating codes into potential themes and gathering all data relevant to each potential theme. We will follow an inductive approach to data analysis to ensure that findings emerge directly from the participants’ narratives and viewpoints, rather than being constrained by pre-existing frameworks or assumptions.

## 5. Results

A study report will be shared with study participants through the same channels that were used to conduct the interviews with them, if they agreed to be further contacted by email for this scope. The study findings will form the basis for one or more open-access scientific publication(s) in a peer-reviewed journal. The outcomes will be also disseminated by means of a policy brief, conference presentations and meetings of NGOs active in assistance to migrants in Flanders or other relevant stakeholders who are involved in migrant assistance, healthcare or administrative affairs in Flanders. The policy briefs and dissemination to stakeholders are key for maximizing the social value of the study, and the likelihood that findings may provide useful guidance to improve the collection and use in research of data on migrants health.

## Authors’ contributions

SA contributed to conceptualisation of the study, article selection, data collection through semi-structured qualitative interviews, data analysis, protocol drafting and critical revisions. LM contributed to article selection, data collection through semi-structured qualitative interviews, data analysis, protocol drafting and critical revisions.

## Ethics review

The protocol was submitted for formal review and approval to the Institutional Review Board (IRB) of the Institute of Tropical Medicine [Approval number 1767/24; Date of approval 02/05/2024].

The study will be carried out according to the principles stated in the Declaration of Helsinki (2013 and any further revisions).

Informed Consent (IC) was obtained prior to interviews. Study participants (adults only) were informed that participation in the study is completely voluntary and that the participant can withdraw from the study at any time without any negative consequences.

The interviewer provided all the information about the study, and went through the Participant Information Sheet together with the participant in advance. The Data Protection Officer contact details were included in the ICF for questions about personal data processing.

## Data Availability

No data are associated with this article. Figshare: Interviews topic guides_ENG (version 2).pdf, https://doi.org/10.6084/m9.figshare.28219859.v1. ^26^ This project contains the following underlying data:
• Interviews topic guides_ENG (version 2).pdf Data are available under the terms of the Creative Commons Attribution 4.0 International license (CC-BY 4.0).
