# Supplementary material for: Mapping data on access to and use of medicines among migrants in Flanders
Source: F1000Res. 2025 Jul 8;14:171. Originally published 2025 Feb 6. [Version 2] doi: 10.12688/f1000research.160320.2 (PMC12338173; doi:10.12688/f1000research.160320.2)
Supplement: Peer review history

# Reviewer report: Reviewer response for version 2

**DOI:** 10.5256/f1000research.181211.r398161

**Published:** 2025-07-25

**Authors:** Stein Monteiro

**Affiliations:** 1 Toronto Metropolitan University, Toronto, Ontario, Canada

1) There is a clear policy problem being addressed by this paper: the under utilization of migrant health data. I think this is an important problem to address but the paper has not shown that this is in fact a problem. Can the authors show that migrant data is being under utilized?

 2) While what the WHO has said is a good guidepost, and this paper is written with that as the main motivating factor, I think the paper should try to show how **mapping**, understanding how **deidentifying and curating**, as well as identifying the **methodological and ethical challenges **will contribute to solving the problem of the underutilization of migrant health data. It might be obvious, but will be useful for the reader to see that in the Rationale.

 3) This sentence is incomplete: "Before such data can be effectively used, ethical implications regarding data collection, deidentification, processing, storage, analysis, and dissemination."

 4) How will the studies in the focused review be analyzed. Are you planning to do a comparison between Flanders and Brussels?

 5) The triangulation process is understandable, although it could be made it a little clearer. However, it is not clear how the results from the triangulation will help the main objectives of the study.

Is the study design appropriate for the research question?

Yes

Is the rationale for, and objectives of, the study clearly described?

Partly

Are sufficient details of the methods provided to allow replication by others?

Yes

Are the datasets clearly presented in a useable and accessible format?

Not applicable

Reviewer Expertise:

Economics, Migration Studies

I confirm that I have read this submission and believe that I have an appropriate level of expertise to confirm that it is of an acceptable scientific standard, however I have significant reservations, as outlined above.

# Reviewer report: Reviewer response for version 2

**DOI:** 10.5256/f1000research.181211.r397484

**Published:** 2025-07-25

**Authors:** Cheng Chow

**Affiliations:** 1 The University of Texas at Austin, Austin, USA

Thank you for the substantial revisions. The manuscript is now clearer and better aligned with the study objectives. The rationale for focusing on Flanders is stronger, and the ethical considerations are more thoroughly addressed.

 A few brief suggestions for further improvement:

 1) Specify whether qualitative data will be coded manually or with software.

 2) Consider describing how diversity across migrant subgroups (e.g., asylum seekers vs. long-term residents) will be accounted for in recruitment.

 These remaining issues are relatively minor and should be straightforward to address. Overall, this is a timely and much-needed contribution to the literature on migration health data infrastructure. Thank you again for your thoughtful work.

Is the study design appropriate for the research question?

Partly

Is the rationale for, and objectives of, the study clearly described?

Partly

Are sufficient details of the methods provided to allow replication by others?

Yes

Are the datasets clearly presented in a useable and accessible format?

Not applicable

Reviewer Expertise:

Migration, Health

I confirm that I have read this submission and believe that I have an appropriate level of expertise to confirm that it is of an acceptable scientific standard.

# Reviewer report: Reviewer response for version 1

**DOI:** 10.5256/f1000research.176202.r368652

**Published:** 2025-03-05

**Authors:** Cheng Chow

**Affiliations:** 1 The University of Texas at Austin, Austin, USA

**Overall**: The authors aimed to map existing datasets on access to and use of medicines among migrant populations in Flanders, Belgium, and assess their feasibility for use in research and monitoring. Given the lack of comprehensive migration health data in Europe, this study has the potential to provide valuable insights into existing data sources and their usability. However, there are several areas that require clarification and improvement, as outlined below.

**1. Tittle**

 The title accurately reflects the study’s focus on mapping datasets but does not fully capture the nature of the study as an initiative rather than an empirical research project. The authors may consider clarifying this distinction to ensure consistency between the study design and its presentation.

**2. Background & Rationale**

 The background section is well-structured and provides a strong rationale for the study. However, there are some areas where additional clarification

 2.1. The introduction spends significant space discussing WHO’s framework on migration and health without directly linking it to the study’s objectives. While the WHO framework provides a relevant backdrop, the authors should ensure that its relevance is explicitly stated. Please consider reducing the length of the WHO framework discussion and making a more explicit connection between the framework and the need for data-driven policy in Belgium.

 2.2. The rationale for focusing on Flanders should be strengthened. While the manuscript mentions Belgium's lack of migrant health data, it would be helpful to explain why Flanders was chosen specifically rather than Belgium as a whole.

**3. Study objectives**

 3.1. While the objectives are well defined, the current objectives mix data mapping, feasibility assessment, and ethical considerations without clearly delineating these as separate but interrelated research components. I would question what sources exist for data mapping, can these sources be used for research for data feasibility, and what are the barriers and possible solutions for ethnical and legal challenges?

 3.2. Objective (b) mentions assessing datasets for *accuracy, completeness, and coherence*, but the methodology does not describe how these aspects will be evaluated. What criteria will be used to assess data completeness and accuracy? Will there be a scoring system or expert validation?

**4. Methods**

 4.1. The exclusion of non-English/non-Dutch sources should be justified. Could relevant data sources exist in French, German, or other languages?

 4.2. Will non-peer-reviewed (grey literature) sources be weighted differently in the analysis?

 4.3. It is unclear how extracted data will be analyzed. Will the authors use a framework approach to categorize datasets?

 4.4.  The description of data gatekeepers is somewhat vague. It is unclear whether they include public health officials, policymakers, or dataset managers.

 The recruitment of migrants does not specify whether different migrant groups (e.g., asylum seekers vs. economic migrants) will be considered. I believe the authors would agree with me that migration status, duration of study and many other factors significantly different across migrant groups.

**5. Data Collection & Analysis**

 5.1. Will interviews be coded manually or using qualitative analysis software (e.g., NVivo)?

 5.2. The GDPR compliance discussion is strong, but there is no mention of additional anonymization or security measures. Given the sensitivity of migrant health data, more emphasis on pseudonymization, encryption, and data storage security would be beneficial.

Is the study design appropriate for the research question?

Partly

Is the rationale for, and objectives of, the study clearly described?

Partly

Are sufficient details of the methods provided to allow replication by others?

Yes

Are the datasets clearly presented in a useable and accessible format?

Not applicable

Reviewer Expertise:

Migration, Health

I confirm that I have read this submission and believe that I have an appropriate level of expertise to confirm that it is of an acceptable scientific standard, however I have significant reservations, as outlined above.

# Response

**Published:** 2025-03-20

**Authors:** Loes Meukens

Thank you for your positive feedback and constructive review. Your comments and suggestions are very helpful in improving our protocol. We appreciate your patience and will revise certain sections and provide responses to your comments by mid-April.

# Response

**Published:** 2025-05-08

**Authors:** Loes Meukens

**Overall: The authors aimed to map existing datasets on access to and use of medicines among migrant populations in Flanders, Belgium, and assess their feasibility for use in research and monitoring. Given the lack of comprehensive migration health data in Europe, this study has the potential to provide valuable insights into existing data sources and their usability. However, there are several areas that require clarification and improvement, as outlined below.**

**1. Title**

**The title accurately reflects the study’s focus on mapping datasets but does not fully capture the nature of the study as an initiative rather than an empirical research project. The authors may consider clarifying this distinction to ensure consistency between the study design and its presentation.**

 We appreciate your feedback and your careful review of our manuscript. However, we respectfully disagree with the suggestion that the study is an initiative rather than an empirical research project. While Stage I involves a focused review of existing datasets, Stage II includes qualitative interviews. This empirical component involves the collection and analysis of primary data and thus qualifies the study as empirical research. 

 To avoid any potential misunderstanding, we have replaced the term *“research initiative”* with *“research study”* in the manuscript text where relevant, while retaining the original title, which we believe accurately reflects the aims of the study.  

**2. Background & Rationale**

**The background section is well-structured and provides a strong rationale for the study. However, there are some areas where additional clarification**

 Thank you for the positive feedback on the structure and rationale of the background section. Below are our replies to your suggestions about the various points that need clarification.  

**2.1. The introduction spends significant space discussing WHO’s framework on migration and health without directly linking it to the study’s objectives. While the WHO framework provides a relevant backdrop, the authors should ensure that its relevance is explicitly stated. Please consider reducing the length of the WHO framework discussion and making a more explicit connection between the framework and the need for data-driven policy in Belgium.**

 Thank you for your suggestion. We revised the text to clearly establish the connection between the WHO framework and the study’s focus on identifying and curating datasets that can inform data-driven policy and healthcare interventions for migrant populations in Belgium. You can find the revised text under section *1.2 Rationale*. 

**2.2. The rationale for focusing on Flanders should be strengthened. While the manuscript mentions Belgium's lack of migrant health data, it would be helpful to explain why Flanders was chosen specifically rather than Belgium as a whole.** 

 Thank you for your comment. We focused on Flanders specifically for pragmatic reasons. These include the language capacity of our research team, and the established network within Flanders, which is more concentrated in this region compared to other parts of Belgium. Expanding the study to include other regions would have introduced significant logistical challenges related to budget and time constraints, which could compromise the feasibility of the study. We acknowledge that this regional focus limits the generalizability of our findings to Belgium as a whole. However, we will clearly reflect on this limitation when discussing the outcomes of our study and outline the implications for future research in other regions of Belgium. We added the following to address your suggestion: 

*“We focused on Flanders due to pragmatic reasons related to the language capacity of our research team, and our established network in the region. Expanding to other regions of Belgium would have introduced logistical challenges, including language barriers, and increased costs and time constraints. We acknowledge that this regional focus limits the generalizability of our findings.”*

***3.*** **Study objectives**

**3.1. While the objectives are well defined, the current objectives mix data mapping, feasibility assessment, and ethical considerations without clearly delineating these as separate but interrelated research components. I would question what sources exist for data mapping, can these sources be used for research for data feasibility, and what are the barriers and possible solutions for ethnical and legal challenges?**

 Thank you for your suggestion. We revised the description of the specific aims of our study to read: 

- *“ A) Data mapping: This objective focuses on identifying existing datasets related to migrant medicine use in Flanders. We will assess the availability and coverage of these sources, considering whether they can be used for research and M&E purposes.  *

- *B) Feasibility Assessment: Our study will evaluate feasibility of applying the sources of data identified by this study in future research studies that aim at evaluating the access of/use of medicines among the diverse migrants population.  *

- *C) Ethical considerations: We will describe the challenges related to accessing and using these data, including ethical and legal challenges, and to develop and describe viable strategies and recommendations for deidentification, data curation and risk mitigation.” *

**3.2. Objective (b) mentions assessing datasets for accuracy, completeness, and coherence, but the methodology does not describe how these aspects will be evaluated. What criteria will be used to assess data completeness and accuracy? Will there be a scoring system or expert validation?**

 For the evaluation of accuracy, completeness, and coherence, we will conduct a focused review of the datasets used in reporting medicine use. This review will help identify any limitations or challenges within the data. We will then discuss these limitations in detail during the qualitative interviews with participants, allowing us to gather insights and validate the findings from the data sources. Through this process, we aim to assess the quality and reliability of the data from both a technical and a practical perspective. 

**4. Methods**

**4.1. The exclusion of non-English/non-Dutch sources should be justified. Could relevant data sources exist in French, German, or other languages?**

 Thank you for referring to this issue. As we addressed in our response to Reviewer 2 in Section 4.1.1, we focused on Dutch and English publications for this study. Dutch is the official language of Flanders and the primary language used in regional governmental and institutional reports. English was included due to its widespread use in academic publishing and its prominence in reports by international and non-governmental organizations. While French and German are also official languages in Belgium, it is less likely that literature specifically addressing migrants' access to healthcare in Flanders would be published exclusively in those languages. 

**4.2. Will non-peer-reviewed (grey literature) sources be weighted differently in the analysis?**

 Yes, we will include grey literature in our focused review. This is mentioned in section 4.1.1.  

**4.3. It is unclear how extracted data will be analyzed. Will the authors use a framework approach to categorize datasets?**

 We added the following under section *“e. Data Extraction and Synthesis”* to address your question: 

*“The data extracted of this focused review will be collated, summarized and reported in a manner that aligns with the aim of the focused review. We will conduct thematic analysis and present its outcomes in a narrative format.”*

**4.4.  The description of data gatekeepers is somewhat vague. It is unclear whether they include public health officials, policymakers, or dataset managers.**

 We agree that further clarification could be helpful. In the revised text, we have now explicitly included representatives of public health offices or institutions among the data gatekeepers.  

**The recruitment of migrants does not specify whether different migrant groups (e.g., asylum seekers vs. economic migrants) will be considered. I believe the authors would agree with me that migration status, duration of study and many other factors significantly different across migrant groups.**

 As noted, our study focuses on migrants who have been living in Belgium for over five years and who are active members of the migrant community. This includes individuals with diverse migration backgrounds—such as former asylum seekers, refugees, or economic migrants—who are currently in leadership roles within community organizations or social groups. While we do not select participants based on their current legal status, we intentionally target those who are well-connected within the community and familiar with the challenges migrants face in accessing medicines. This approach allows us to gather informed insights while capturing a range of experiences across different migrant groups. 

**5. Data Collection & Analysis**

**5.1. Will interviews be coded manually or using qualitative analysis software (e.g., NVivo)? ** 

 Thank you for this question. The interviews will be coded manually, using Microsoft Word.  

**5.2. The GDPR compliance discussion is strong, but there is no mention of additional anonymization or security measures. Given the sensitivity of migrant health data, more emphasis on pseudonymization, encryption, and data storage security would be beneficial.**

 Thank you for your suggestion. We have expanded the manuscript as follows to include a more detailed explanation of the anonymization and data security measures that will be taken throughout the study: 

*“In the Stage I of the study, when performing the focused review, no personal data will be processed. For the Stage II, when conducting the interviews, the lawful ground for the processing of the participant personal data will be the written consent of the participant. The study will also be entered in the Institute of Tropical Medicine GDPR registry of personal data processing activities and the requirements are reviewed by the Data Protection Officer. All study data will be pseudonymized at the earliest convenience. Identifiers are retained at a separate and secure location, and deleted (full anonymization) after the study is published. Identifiable information in any audio or videocall recording will also be minimized to the extent possible.”*

# Reviewer report: Reviewer response for version 1

**DOI:** 10.5256/f1000research.176202.r368648

**Published:** 2025-03-03

**Authors:** Yu par Khin

**Affiliations:** 1 Institute of Science Tokyo, Tokyo, Japan

Thank you for giving me opportunity to review this article. This protocol aims to map available data sources on migrants' access to medicines in Flanders, Belgium. I think this is a great effort and would have scientific impact. I have a few comments on the following topics.

 Abstract

 1. Objective

 This study is to map the available datasets about access to and use of medicines. I am wondering is it the prescription based medical access to OTC medication?

 2. Keywords

 I think "access" is one of the keywords.

 Introduction

 1. I understand that data protection is important to protect the migrant population. It would be great if the introduction can add the following information.

 1.a. How people access medicine in Belgium and other EU countries and why migrants can be left behind?

 Methods

 4.1.1. b. Do you mean literature published in English and Dutch? Why do you particularly choose these two languages but not others like German or French?

 4.1.3. It would be great if you can describe more elaboratively on how the qualitative interviews be compared with the finding from Stage I. On which part and how? You should also add which kind of data will be collected from two groups of participants in 4.3. Then readers can clearly understand how these data from Stage I and II will be comparable.

 4.2.1. Are community members only first generation migrants?

 4.2.3 Please briefly describe how will you approach the community members and data gatekeepers separately?

 4.3. What about migrants who cannot speak Dutch or English? Will you include interpreters?

 4.4. Will you use deductive or inductive approach? If you use deductive approach, on which framework will you based the analysis on?

Is the study design appropriate for the research question?

Yes

Is the rationale for, and objectives of, the study clearly described?

Yes

Are sufficient details of the methods provided to allow replication by others?

Partly

Are the datasets clearly presented in a useable and accessible format?

Not applicable

Reviewer Expertise:

Access to healthcare by migrant, Qualitative research, Systematic review

I confirm that I have read this submission and believe that I have an appropriate level of expertise to confirm that it is of an acceptable scientific standard, however I have significant reservations, as outlined above.

# Response

**Published:** 2025-05-08

**Authors:** Loes Meukens

**Thank you for giving me opportunity to review this article. This protocol aims to map available data sources on migrants' access to medicines in Flanders, Belgium. I think this is a great effort and would have scientific impact. I have a few comments on the following topics.**

 We appreciate your thoughtful review of our protocol. Your comments and suggestions have been very helpful in improving our protocol. Below, we respond to all comments. 

**Abstract**

**1.Objective**

**This study is to map the available datasets about access to and use of medicines. I am wondering is it the prescription based medical access to OTC medication?**

 Thank you for this question. Both prescription based medication and OTC medication are of interest in this study.  

**2. Keywords**

**I think "access" is one of the keywords.**

 Thank you for this observation. We added “access” to the key words.  

**3. Introduction**

**1. I understand that data protection is important to protect the migrant population. It would be great if the introduction can add the following information.**

**1.a. How people access medicine in Belgium and other EU countries and why migrants can be left behind?**

 Thank you for this suggestion. We have added the following to address you suggestion: 

*“Access to essential medicines for migrants in Europe varies significantly across countries due to differences in healthcare systems, legal frameworks, and the socio-political status of migrants. Migrants, particularly asylum seekers, refugees, and undocumented migrants, often face substantial barriers to accessing medicines. These barriers include limited legal entitlements, financial constraints, language issues, and lack of familiarity with healthcare systems. In many EU countries, asylum seekers and undocumented migrants may be excluded from national health insurance systems or face long waiting periods for coverage, which can delay or restrict access to essential medications. In Belgium, asylum seekers are entitled to medical care under the "Aide Médicale Urgente" (AMU) system, which covers urgent and necessary healthcare, including some essential medicines. However, this provision often does not cover the full range of chronic disease treatments. While some NGOs and local health actors provide support to fill these gaps, persistent challenges remain, underscoring the need for more inclusive and coordinated policies and better data to ensure equitable access to medicines for all migrant populations.”*

**Methods**

**4.1.1. b. Do you mean literature published in English and Dutch? Why do you particularly choose these two languages but not others like German or French?** 

 We focused on Dutch and English publications. Dutch is the official language of Flanders, making it the primary language for regional governmental and institutional reports. English was included due to its widespread use in academic publishing and its prominence in reports by international and non-governmental organizations. While French and German are also official languages in Belgium, it is less likely that literature specifically addressing migrants’ access to healthcare in Flanders would be published exclusively in those languages.  

**4.1.3. It would be great if you can describe more elaboratively on how the qualitative interviews be compared with the finding from Stage I. On which part and how?**

 We added the following to address your suggestion: 

*“To enable meaningful triangulation, we will compare the types and characteristics of data sources identified in Stage I (e.g., scope, accessibility, population coverage, variables collected) with insights gathered in Stage II about their practical use, availability, and limitations as described by the participants. In particular, we will assess whether data sources identified in literature are also recognised or used by key stakeholders in Flanders, and whether additional data sources are known to local actors but were reported in the literature. We will also compare reported legal and ethical considerations across both stages. This comparison will help to validate, expand, or challenge the findings from the literature and identify gaps between formal reporting and actual data access and usage practices in the field. The compared and contrasted outcomes of both studies will be presented and discussed with a research advisory group, to broaden the understanding of the outcomes and formulate recommendations for further research. Table 1 lists a summary of the different stages that will take place during the study and the respective methods for data collection.”*

**You should also add which kind of data will be collected from two groups of participants in 4.3. Then readers can clearly understand how these data from Stage I and II will be comparable.** 

 We added the following to section 4.3: 

*“The interviews will collect two types of data. From data gatekeepers, we will collect information on the availability, accessibility, structure, content, and quality of existing data sources relevant to access to and use of medicines among migrants in Flanders, as well as legal, technical, and ethical considerations in using these data. From members of migrant communities, we will collect insights on whether and how their access to medicines may be represented (or overlooked) in existing data sources, including perceptions of data use, trust in institutions, and barriers to inclusion.”*

**4.2.1. Are community members only first generation migrants?**

 Thank you for raising this point. In the text we describe community members as migrants who have been living in Belgium for over 5 years and are active members of the community. These include individuals with migration background who are in leadership roles within community organizations and social clubs. This definition does not only apply to first-generation migrants but to all who identify as migrants. 

**4.2.3 Please briefly describe how will you approach the community members and data gatekeepers separately?**

 We added the following to section 4.2.3 to explain how we approached the data gatekeepers and community members: 

*“Data gatekeepers will primarily be approached through institutional networks, professional contacts, and health sector organizations. In contrast, community members will be approached via civil society organizations, community-based networks, and local NGOs working with migrant populations”*

**4.3. What about migrants who cannot speak Dutch or English? Will you include interpreters?**

 Thank you for pointing out the need for clarification. In this case we will include interpreters to ensure that migrants who do not speak Dutch or English can fully participate in the study. 

**4.4. Will you use deductive or inductive approach? If you use deductive approach, on which framework will you based the analysis on?**

 We are using an inductive approach to our analyses. We added the following to explain this point: 

*“We will follow an inductive approach to data analysis to ensure that findings emerge directly from the participants’ narratives and viewpoints, rather than being constrained by pre-existing frameworks or assumptions.”*

# Reviewer report: Reviewer response for version 1

**DOI:** 10.5256/f1000research.176202.r366029

**Published:** 2025-02-17

**Authors:** Alejandro Gil-Salmerón

**Affiliations:** 1 International Foundation for Integrated Care, Oxford, UK

The study is presented in a timely manner to understand the medication access needs of immigrant people in Belgium.

 In the first paragraph, it discusses health data and the migrant population at an international level. The last sentence needs theoretical support. The review conducted by Lebano et al. (2020) *Migrants’ and refugees’ health status and healthcare in Europe: a scoping literature review* concludes that the data is fragmented, making it difficult to reach conclusions due to the diversity of studies.

 Regarding the review for mapping databases, I do not believe this is a research objective but rather a step in the process of conducting the research. In this sense, a research objective would be to study these databases by defining, for example, their size, quality, and the type of data collected. To achieve this, it will be necessary to map the databases following a rigorous scientific methodology (e.g., systematic review). The second objective would be ideal for covering both aspects.

 Regarding interviews with key informants, we must consider that key informants are those who can provide key information from a specific perspective, but within qualitative research, they are not part of the study population. Therefore, the study population should be better defined as those who use these data and need access to them.

Is the study design appropriate for the research question?

Partly

Is the rationale for, and objectives of, the study clearly described?

Yes

Are sufficient details of the methods provided to allow replication by others?

Partly

Are the datasets clearly presented in a useable and accessible format?

Not applicable

Reviewer Expertise:

Access to healthcare, migration health, disadvatanged groups, integrated care, populations health

I confirm that I have read this submission and believe that I have an appropriate level of expertise to confirm that it is of an acceptable scientific standard, however I have significant reservations, as outlined above.

## References

[1] : Migrants' and refugees' health status and healthcare in Europe: a scoping literature review. *BMC Public Health* .2020;20(1) : 10.1186/s12889-020-08749-8 1039 10.1186/s12889-020-08749-8 32605605 PMC7329528

# Response

**Published:** 2025-05-08

**Authors:** Loes Meukens

**1. The study is presented in a timely manner to understand the medication access needs of immigrant people in Belgium.**

 Thank you for your positive feedback. Your comments and suggestions have been very helpful in improving our protocol. Please find below our point-by-point responses to your comments and suggestions.  

**2. In the first paragraph, it discusses health data and the migrant population at an international level. The last sentence needs theoretical support. The review conducted by Lebano et al. (2020) Migrants’ and refugees’ health status and healthcare in Europe: a scoping literature review concludes that the data is fragmented, making it difficult to reach conclusions due to the diversity of studies.**

 Thank you. We added the following to address your suggestion: 

 “However, the collection of such data remains fragmented across settings and time periods, often lacking standardised and shared procedures. This impedes efforts to monitor and improve migrant health in Europe. As Lebano et al. (2020) concluded in their scoping review, the lack of reliable and routine data collection, inconsistent definitions of migrant categories, and variations in national health systems all present major barriers to assessing and comparing health outcomes. While interest in migrants’ health is growing, these limitations create serious challenges in developing evidence-based policies and tailored healthcare interventions” 

**3. Regarding the review for mapping databases, I do not believe this is a research objective but rather a step in the process of conducting the research. In this sense, a research objective would be to study these databases by defining, for example, their size, quality, and the type of data collected. To achieve this, it will be necessary to map the databases following a rigorous scientific methodology (e.g., systematic review). The second objective would be ideal for covering both aspects.**

 Thank you for your suggestion. We have revised the section that lists the specific aims  as follows: 

 “To study different quantitative and/or qualitative data sources that contain information on access to and use of medicines by defining their size, quality,  and the type of data collected, and understand how they can be de-identified, curated, and assessed for accuracy, completeness and coherence, as well as comparability across datasets, for being used for M&E or research.” 

**4. Regarding interviews with key informants, we must consider that key informants are those who can provide key information from a specific perspective, but within qualitative research, they are not part of the study population. Therefore, the study population should be better defined as those who use these data and need access to them.**

 Thank you for your insightful comment. We appreciate your point about the distinction between key informants and the study population. We would like to clarify that, in the context of our study, the individuals we refer to as key informants are those responsible for the collection, management, and processing of datasets related to access to or use of medicine. These key informants are integral to answering our research questions. They will provide crucial information that directly aligns with our study's aims. While they are not the direct beneficiaries of the data, their role in providing the necessary insights makes them central to the study.
